# Is the Thin Bronchoscope the Right Compromise Between Ultrathin and Conventional Bronchoscopy for Peripheral Pulmonary Lesions (PPLs)? A Retrospective Study

**DOI:** 10.3390/jcm14113855

**Published:** 2025-05-30

**Authors:** Filippo Lanfranchi, Gioele Castelli, Laura Mancino, Gabriele Foltran, Lucio Michieletto

**Affiliations:** 1 Respiratory Disease Unit, Department of Cardiac Thoracic and Vascular Sciences, Ospedale dell’Angelo, 30174 Venice, Italy; 2 Respiratory Disease Unit, Department of Cardiac, Thoracic, Vascular Sciences and Public Health, University of Padova, Via Giustiniani 2, 35128 Padova, Italy

**Keywords:** thin bronchoscope, lung cancer, peripheral pulmonary lesion(s), R-EBUS

## Abstract

**Background/Objectives:** Peripheral pulmonary lesions (PPLs) are the current challenge in bronchoscopy. Novel endoscopic approaches allow us to reach PPLs better than a few years ago. In patients with resectable non-small cell lung cancer (NSCLC), perioperative chemotherapy is associated with significantly greater event-free survival; this means that histological assessment before the resectable surgery of PPLs is becoming mandatory. Our objective was to evaluate the diagnostic yield (DY) of a thin bronchoscope (TB) for PPLs suspected for lung cancer that are not reachable with conventional bronchoscopy. **Methods:** A total of 176 patients with PPLs were evaluated from January 2022 to July 2023. Of the patients, 26 presented with not reachable PPLs with conventional bronchoscopy, and underwent the procedure again with a TB. When possible, R-EBUS was used. PPLs’ dimensions were recorded via chest computed tomography (CT) scan. DY was evaluated. **Results:** Mean lesion size was 29 mm, and overall DY for TB was 65% (17/26). When the lesion was bigger than 20 mm, DY was 76.5% (13/17), whereas in lesions smaller than 20 mm, DY was 55% (5/9). When PPLs presented a bronchus sign in the CT scan, diagnostic performance of TB was significantly better (76.5% vs. 40%, p = 0.04) compared to PPLs without a bronchus sign, independent from PPL dimensions. R-EBUS did not change DY. **Conclusions:** TB easily allows us to reach and sample PPLs with a high DY if a bronchus sign is positive, independently from PPL dimensions. Further studies are needed to evaluate if more flexible and penetrating bronchial wall biopsy tools can augment DY for PPLs with TB.

## 1. Introduction

Lung cancer is the most fatal and the second most common cancer worldwide. A lung nodule can represent the earliest detectable stage of lung cancer, defined as a peripheral pulmonary lesion (PPL). It has been well demonstrated that the stage of diagnosis is inversely related to prognosis, with early detection leading to significant improvements in survival [1].

According to randomized controlled trials, the use of a diagnostic chest computed tomography (CT) scan and the implementation of lung cancer screening reduces mortality in a high-risk cohort patient. In fact, both the National Lung Screening Trial (NLST) and the NELSON trial have shown reduced lung cancer mortality with low-dose CT screening compared to chest radiography or no screening [2,3].

Consequently to these screening programs, the number of pulmonary nodules detected yearly continues to increase. As is obvious, the demand for tissue sampling is increasing in parallel. However, conventional strategies have a low diagnostic rate in regard to PPLs. Therefore, novel bronchoscopy approaches have been developed, such as radial-probe endobronchial ultrasound (R-EBUS), fluoroscopy, electromagnetic navigational bronchoscopy (EMN), virtual navigational bronchoscopy (VBN), and robotic bronchoscopy (RB). All these techniques expanded the arsenal in the hands of interventional pulmonologists (IP) to avoid CT-guided biopsies of PPLs [4]. Furthermore, new bronchoscopy tools have also been implemented and invented in recent years. Needles for transbronchial needle aspiration (TBNA), conventional biopsy forceps, mini forceps, and cryoprobes are now available to gain more tissue during bronchoscopy procedures. This necessity drives the need to research additional mutational and immunological variations to guide lung cancer therapy. All the new molecular analysis performed with next-generation sequencing (NGS) associated with conventional immunohistochemistry (IHC) for markers such as programmed-death ligand 1 (PDL-1), epidermal growth factor receptor analysis (EGFR), anaplastic lymphoma kinase (ALK), and reactive oxygen species (ROS) raised the demand for larger samples from the pathologists [5].

The necessity for pathological samples to perform pre-operative molecular and IHC diagnosis arises also from the possibility to perform target neo-adjuvant therapy. Recent trials, such as the CheckMate 816 trial, have shown the importance of a correct diagnosis even in resectable non-small cell lung cancer (NSCLC) [6].

The above-mentioned novel bronchoscopic approaches are not widespread and are associated with logistical and economic issues. These techniques (VBN, EBN, RB), and some sampling tools, such as cryoprobes, are very expensive, and need a specific setting to be performed.

The need for anesthesiology assistance due to rigid bronchoscopy approaches, alongside the need for post-operative assessment, greatly reduces the possible number of daily procedures. On the other hand, TB can be used in a conventional bronchoscopy setting and without the need for airway control and general anesthesia.

Usually, transbronchial lung biopsies (TBLBs) for PPLs are performed with conventional bronchoscopy (CB) (outer diameter–OD–of 5.8 mm, working channel of 2.8 mm, usually). The use of a thin (TB) or ultrathin bronchoscope (UTB), which are instruments that are smaller compared to CB for PPLs, is primarily aimed at improving DY due to the better accessibility to small, distal airways.

The reasons to use a TB/UTB lies in fact that the major limitation of CB is the anatomic constraints of the physical bronchoscope and its inability to reach distal subsegmental levels owing to the bronchoscope’s large outer diameter. Conversely, a TB/UTB (OD < 3 mm) can go deeper into the lung periphery, often reaching the ninth bronchial generation, gaining improved access to peripheral lesions for tissue sampling [7]. A TB/UTB is often combined with other guided techniques, such as CT-guidance, virtual bronchoscopic navigation (VBN) and R-EBUS, to improve lesion localization [4,7].

Compared to CB performed with larger scopes, the use of a TB can represent a good compromise between the possibility to reach distal airways and the complication management that is difficult with the small operative channel of a UTB.

In the literature, a study comparing a TB with R-EBUS to CB with fluoroscopy found that the DY was higher in the thin bronchoscope group (49% vs. 37%), although this difference was not statistically significant and had a DY that was still too low [8]. One study compared a TB with CB with no differences in terms of DY [9]; none of these studies evaluated if a TB utilized subsequently to CB can improve DY.

### Aim of the Study

The aim of the present study is to evaluate the DY of a TB for PPLs suspected of lung cancer that is not reachable with CB.

## 2. Materials and Methods

### 2.1. Study Design and Population

In the present study, we retrospectively enrolled all the patients who underwent CB for PPL suspected of lung cancer from January to July 2023 in Ospedale dell’Angelo, AULSS3 Serenissima, Venice-Mestre, Italy. CB was performed with a therapeutic bronchoscope with a 5.8 mm OD and a 2.8 mm working channel (Fuji 580T, Fujifilm, Tokyo, Japan). The procedures were performed by IP in our Pulmonology Unit (Ospedale dell’Angelo, AULSS3 Serenissima, Venice-Mestre, Italy). Every patient underwent a chest CT scan and signed a written consent form before the procedure. If the nodule was not reachable with CB, the patient repeated the procedure with a TB (4.1 mm outer diameter, 2.0 mm working channel-EB-710P, Fujifilm, Tokyo, Japan).

In the patients who underwent a second bronchoscopy with a TB, demographics, and PPL dimensions (in its narrowest diameter) in chest CT scans were recorded. Also, biopsy tools, number of passes, and use of R-EBUS were recorded. DY was evaluated.

The patients were divided in two groups: diagnostic and non-diagnostic.

A diagnostic sample was defined as a sample that was positive for cancer, and DY was defined as the ratio between true-positive plus the true-negative samples and the totality of patients in which a TB was used.

Furthermore, all complications were recorded (respiratory failure, infections, pneumothorax/pneumomediastinum, hemoptysis, death, cardiovascular disorders, hemodynamic instability).

### 2.2. Thin Bronchoscopy (TB) Procedure

**Figure 1:**
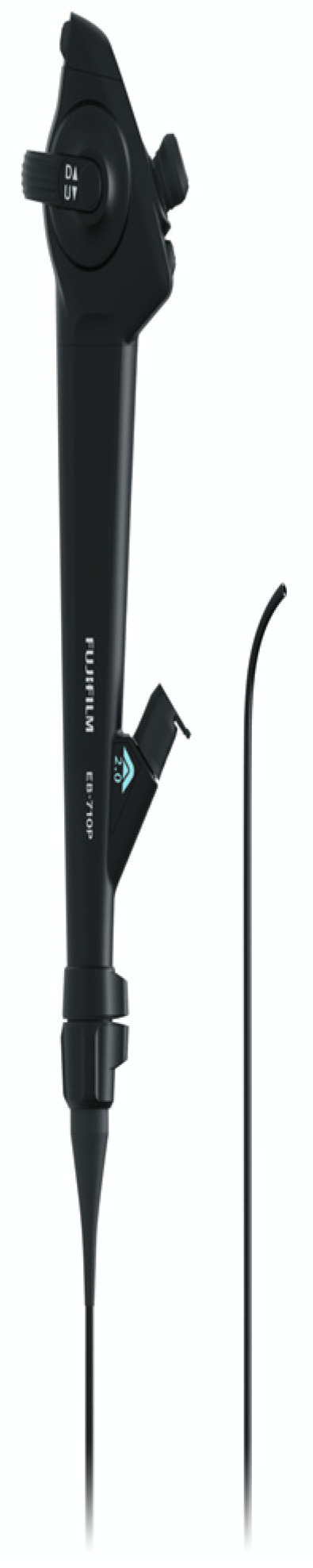
Presentation of the Fuji EB-710P (Fujifilm, Tokyo, Japan) bronchoscope used for the TB procedures of the study (reprinted with Fujifilm permission).

**Figure 2:**
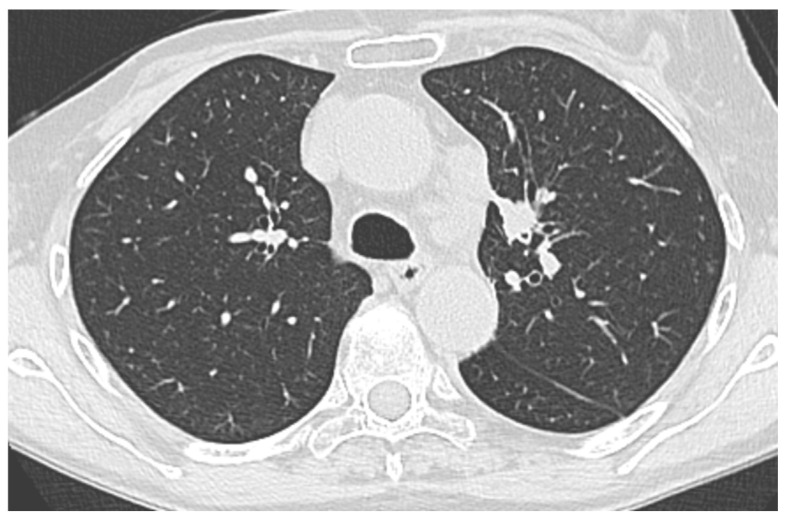
Chest CT scan example of a peripheral pulmonary lesion (PPL) sampled with a thin bronchoscope (TB).

**Figure 3:**
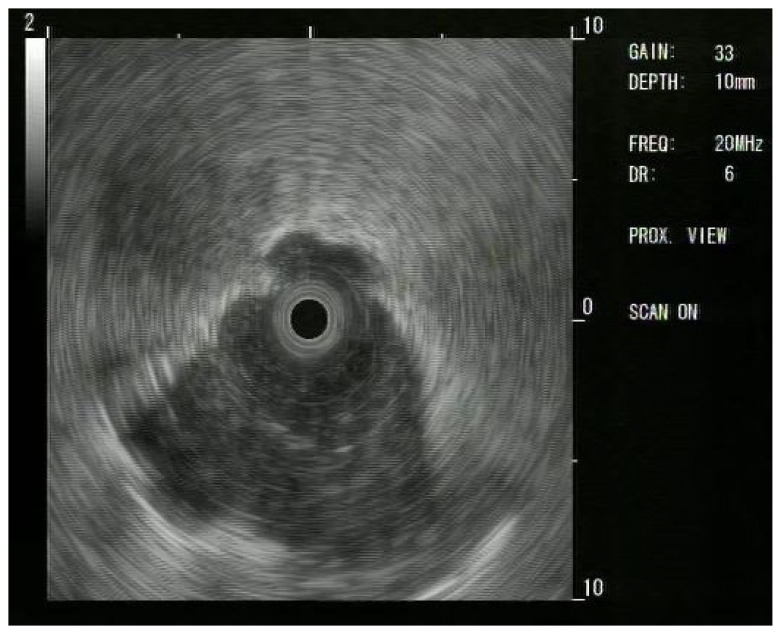
Radial EBUS (R-EBUS) confirmation of a PPL; the use of R-EBUS helps the procedure guiding the transbronchial biopsy. The consolidative pattern in the lower part of the image confirms that a nodule is outside the bronchus.

Bronchoscopy with a TB was performed under moderate sedation using our institution’s standard protocol. Patients were monitored during all parts of the procedure and sedation was titrated to the patient’s comfort and ability to follow commands. A TB was used (EB-710P, Fujifilm, Tokyo, Japan), shown in Figure 1. Target tool identification was used at the discretion of the bronchoscopist, depending on the endoscopic finding and chest CT scan imaging (R-EBUS, R-EBUS + fluoroscopy, none), shown in Figure 2 and Figure 3. The target lesion was sampled with 2.00 mm forceps (Endojaw FB-211D, 1.9 mm, Olympus Medical Systems Corp., Tokyo, Japan) or with a conventional TBNA needle (Wang^®^ Transbronchial cytology needle MW-122, Conmed, Utica, NY, USA).

Quality and number of specimens were based, if applicable, on rapid-on-site evaluation (ROSE) [10,11], by using Diff-Quik stain, to guide the number and site of the next biopsies. All ROSEs were performed by the pulmonologist team. The samples obtained were processed for cytological (if a slide was performed) and histological evaluation; all pathological diagnoses were performed by pathologists at the Venice-Mestre Hospital, Pathologic Anatomy Unit. If the sample was adequate, complete immunohistochemistry (IHC) or next-generation sequencing (NGS) (where indicated) were performed.

### 2.3. Ethic Statement

This was a retrospective study on anonymized patient data collected from electronic medical records. This study was approved by the Ethics Committee of the University of Padua (number 21482; approved on 19 January 2023).

Consent for retrospective evaluation of data was waived in accordance with local legislation.

The work described has been carried out in accordance with The Code of Ethics of the World Medical Association (Declaration of Helsinki). All patients signed a written consent form before the procedure.

### 2.4. Statistical Analysis

Categorical variables are expressed as absolute (n) and relative values (%) whereas continuous variables are expressed as median and ranges. To compare diagnostic and non-diagnostic procedures, Chi square test and Fisher’s exact test for categorical variables and Mann–Whitney U test for continuous variables were used, as appropriate. DY was defined as the ratio between true-positive plus the true-negative samples and the totality of patients in which a TB was used.

All data were analyzed using GraphPad Prism 7.0 (GraphPad Software Inc., La Jolla, CA, USA). *p*-values < 0.05 were considered statistically significant.

## 3. Results

We identified 26 patients who underwent bronchoscopy with a TB after a CB to biopsy PPLs that were not reachable with a standard bronchoscope size between January and December 2022; descriptive statistics of patients and PPLs are shown in Table 1.

A TB procedure was performed in 26 patients, with a DY of 65% (17/26). In the two groups (diagnostic vs. non-diagnostic), there were no statistically significant differences in terms of age, sex, PPL dimensions and number of passes (Table 1).

In the non-diagnostic group, 7/9 nodules were located in upper lobes (one nodule in LB1, two nodules in RB3, two nodules in RB1, one nodule in RB2, one nodule in LB3), one was in the middle lobe (RB5) and one was in the lower lobe (LB8).

In the diagnostic group, 13/17 were located on the upper lobes (three nodules in RB4, one nodule in RB3, five nodules in RB1, two nodules in LB1, two nodules in RB4) and four in the lower lobes (two nodules in LB9, one nodule in RB9, one nodule in RB6).

Interestingly, there were also no statistically significant differences between the two groups regarding R-EBUS use and if the nodule was bigger than 20 mm in diameter; moreover, the use of a TBNA needle or biopsy forceps did not change the DY, whereas the presence of a bronchus sign in the chest CT was significantly more present in the diagnostic group (*p* = 0.04, Table 2).

A total of 17 cancer diagnosis were obtained, 11 of them were non-small cell lung cancer (NSCLC): 8 adenocarcinomas (ADKs), 3 squamous cell carcinomas (SCCs), 4 carcinomas not otherwise specified (NOS), 1 metastasis from breast cancer and 1 typical carcinoid. In the diagnostic group, according to the pathological report, the sample was adequate for PDL-1 analysis in 11/15 patients (two patients of the diagnostic group had typical carcinoid and breast cancer metastasis).

The request for NGS, which was made by oncologists after discussion in a multidisciplinary tumor board (MDT), as requested by Our Regional Legislation, was performed in 8/17 samples, and in four of them (50%) the amount of tissue was sufficient for the complete genetic profiling of the cancer.

Regarding adverse events, there were no patient-related complications observed: in particular, no complications were observed in terms of pneumothorax, mediastinitis, massive hemoptysis, respiratory failure, hemodynamic instability, and cardiac adverse events. Moreover, there was no TB damage during procedures, and none of the patients requested the interruption of the procedure due to intolerance.

## 4. Discussion

Diagnosis of PPL is the current challenge in bronchoscopy and interventional pulmonology, leading to early lung cancer diagnosis; nevertheless, almost 25% of PPLs remain undiagnosed. Bronchoscopy with guide systems, VBN, EMN, robotic bronchoscopy, and transparenchymal nodule approaches (BTPNAs) tend to reach PPLs in higher percentages compared to CB. However, the diagnostic yield actually rarely exceeds 75% [4].

TBs are more efficient than CB to reach PPLs, and this study demonstrates that using a TB, after failed CB attempts, increases DY.

In the literature, it is well recognized that the use of guidance systems has increased the DY for what concerns PPLs: in fact, traditional trans bronchial lung biopsy (TBLB), guided only by fluoroscopy, has historically had a low DY in PPLs, with diagnostic rates for nodules under 2 cm estimated to be 34% and 63% for lesions over 3 cm [12].

Since these results, Ips have moved to using R-EBUS: a thin, flexible catheter with a rotating ultrasound transducer that produces a 360-degree (“radial”) image; the catheter easily passes through the working channel of the scope. This provides a 360-degree view in a 2D plane [12].

In 2011, a meta-analysis of R-EBUS-guided bronchoscopy with 1420 patients reported a pooled diagnostic sensitivity of 73%. Complication rates were similar to those of non-guided bronchoscopy, with a pneumothorax rate of 1%, with less than half of those requiring chest tube placement [13].

A larger and more recent meta-analysis assessing R-EBUS for PPL diagnosis in 2017 found an overall weighted DY of 70.6%. DY was higher in nodules > 2 cm, malignant nodules, and those with a positive bronchus sign [14]. The major limitations in R-EBUS procedures are misinterpretations of radial ultrasound signals and the fact that the radial probe is more flexible than the majority of biopsy tool instruments; thus, after the lesion is identified, the forceps could not reach the target because of its stiffness. Moreover, R-EBUS, contrary to linear EBUS, does not provide a real-time biopsy because the R-EBUS probe has to be withdrawn to allow the biopsy tool insertion in the same working channel of the scope.

Ultrathin bronchoscopes (UTBs), typically with an OD of 3.0 mm and a working channel of 1.7 mm, offer superior maneuverability and can reach more distal bronchi compared to a TB. This enhanced access is particularly beneficial for evaluating PPLs, which are often located in the outer third of the lung [7,8,9,10,11,12,13,14,15,16].

In the literature, several studies have demonstrated that UTBs provide a higher DY for PPLs. For instance, a randomized trial found that the DY was significantly higher with a UTB (70.1%) compared to a TB (58.7%) [7]. Another study reported a DY of 74% with a UTB, compared to 59% with a TB [15]. A meta-analysis also supported these findings, indicating a pooled DY of 65% for a UTB [16].

The use of a UTB is often combined with multimodal devices such as radial endobronchial ultrasound (R-EBUS), VBN, and fluoroscopy to further enhance diagnostic accuracy and localization of lesions [15,17,18]. These combinations allow for real-time imaging and precise targeting of lesions, which is crucial for obtaining adequate biopsy samples.

The differences between a UTB and a TB are several: regarding DY, a UTB seems to provide a higher DY compared to a TB [7,15]. Regarding specimen size, TBs have a larger working channel (generally 2.0 mm) compared to UTBs (working channel of 1.7 mm), allowing for the use of larger biopsy instruments and potentially larger specimen sizes. Moreover, regarding the compatibility with other tools, TBs are more compatible with a wider range of instruments, including dedicated guide sheaths (GSs), larger forceps, and aspiration needles, which facilitate repeated sampling from the same location. UTBs have limited compatibility due to their smaller working channel, which can restrict the types of instruments used, and are less adequate to manage potential complications such as mucus plugging or bleeding; moreover, the smaller outer and inner diameters of UTBs consequently do not allow a satisfactory endoscopic view [7]. Finally, UTBs often require the integration of multiple imaging modalities such as those described above, consequently increasing the complexity and duration of the procedure.

The present study confirms that a TB is a useful tool to increase DY over the CB, without recurring with additional guidance systems (i.e., VBN, EMN, cone-beam CT-CBCT) that necessarily increases the time of the procedure, often also necessitating general anesthesia or deep sedation, with an anesthesiologist’s involvement.

In the literature, a study performed by Nishii Y et al. [19] showed an increased DY if the TB was substituted with a UTB: in this paper, the patients underwent a procedure with a TB plus R-EBUS and, if the probe was not within the lesion (i.e., adjacent or no visible lesion), the TB was substituted with a UTB, with an increased DY. In that study, the presence or not of the bronchus sign was statistically significant between diagnostic and non-diagnostic bronchoscopy, but both procedures (TB and UTB) were performed with the help of a guidance system (VBN) [19]. In another RCT by Oki M., the overall diagnostic yield was significantly higher in the UTB group than in the TB group (70.1% vs. 58.7%, respectively; *p* = 0.027). The procedure duration was significantly shorter in the UTB group (median, 24.8 vs. 26.8 min, respectively; *p* = 0.008). The complication rates were 2.8% and 4.5%, respectively (*p* = 0.574); also, in this paper, VBN was used in both cases (TB and UTB) [7].

Moreover, in another study by Oki M. et al., a UTB presented a higher DY compared to a TB with the use of a guide sheath (TB-GS): in fact, the UTB group presented a statistically higher DY but, again, a UTB was used with VBN [15].

In all the above-mentioned studies, VBN as a guidance system was used, over conventional R-EBUS and fluoroscopy, whereas in our study, no additional guidance systems over R-EBUS and fluoroscopy were necessary; moreover, in the present study, a different brand of TB was used, and no data for this are available in the literature, to the best of our knowledge.

In our study, the use of a TB allowed us to reach and diagnose 17/26 (=65%) PPLs that were not reachable with CB, with no additional guidance systems but just performing bronchoscopy twice, with the same tools, increasing the overall DY for PPLs.

The presence of the bronchus sign, as confirmed in the above-mentioned studies, statistically increases DY, independently from PPL size; this suggests that, if R-EBUS can reach the lesion, the biopsy tool will also probably reach it.

Recently, the use of flexible and smaller (1.1 mm) cryoprobes (ErbeCryo^®^2, Erbe Elektromedizin GmbH 2023, Tuebingen, Germany) for novel biopsy sampling seems to be a very promising tool to increase DY for PPLs. Their use, moving from interstitial lung diseases (ILDs) to PPL diagnosis, represents a very interesting tool to improve DY of every bronchoscopic procedure; moreover, their flexibility (and ability to collect larger-size samples) can potentially allow us to reach every PPL because of their lower stiffness compared to TBNA needles and forceps, since it is well-recognized that two of the main limiting factors for PPL diagnosis are the biopsy tools and their stiffness [4].

A UTB allows for better accessibility to peripheral bronchi. The 1.1 mm ultrathin cryoprobe can be used through this working channel to perform cryobiopsies. This combination has been shown to improve the DY for PPLs. Specifically, the use of a UTB combined with a cryoprobe has demonstrated a higher R-EBUS localization rate (96.0% vs. 44.6%) and a higher DY (54.0% vs. 30.1%) compared to a TB with a guide sheath (GS) [18]. A TB facilitates the use of a GS for repeated sampling from the same location. The 1.1 mm cryoprobe can also be used with this setup, although the DY is generally lower compared to the UTB setup. However, the TB with GS allows for better control of the biopsy site and can be advantageous in certain clinical scenarios [18]. The larger and artifact-free samples obtained via cryobiopsy improve diagnostic accuracy compared to traditional forcep biopsy. Additionally, the use of cryoprobes has been associated with a favorable safety profile, with manageable bleeding complications and a low incidence of pneumothorax [20].

The most recent paper regarding cryobiopsy and UTBs was written by Oki M. et al., in which the use of cryobiopsy with a UTB was reported as effective, feasible and sufficiently safe for PPL diagnosis [20], always with the help of VBN, with a DY of 74%.

This field needs to be further investigated to evaluate whether cryobiopsy with a TB can be feasible, safe and as effective as when used with a UTB, since with a TB we did not encounter any complications in terms of bleeding, pneumothorax, hemodynamic instability, of respiratory failure; this suggests that a TB is probably less traumatic and more specific for PPLs compared to CB, but it would be interesting to evaluate, in the future, if the same considerations can be applied with TBs and cryobiopsies, without additional guidance systems.

Finally, in the present study, the use of ROSE has guided the number of passes, because it is well recognized that the use of ROSE increases the DY.

Several studies have demonstrated the efficacy of ROSE in improving diagnostic outcomes during TBLB. For instance, Wang et al. found that the diagnostic yield for peripheral lung cancer was significantly higher in the ROSE group compared to the non-ROSE group (42.9% vs. 30.7%, *p* < 0.05) [21]. Similarly, Xu et al. reported that the DY in the use of R-EBUS combined with ROSE group was 85.9%, significantly higher than the 70.3% yield in the R-EBUS without ROSE group (*p* = 0.016) [11]. Moreover, ROSE has been shown to reduce the number of biopsies needed and the overall procedure time. Xu et al. noted that the operation time was significantly shorter in the ROSE group compared to the non-ROSE group (24.6 ± 6.8 min vs. 32.4 ± 8.7 min, *p* = 0.001) [11]. Additionally, the American College of Chest Physicians has highlighted that ROSE can decrease the number of needle passes and reduce the need for additional diagnostic procedures [22], even if these recommendations are referred to linear EBUS (L-EBUS). Given the fact that the implementation of ROSE during TBLB enhances DY, reduces procedure time, and minimizes the number of biopsies required, it would be interesting to evaluate if the use of ROSE during a TB procedure and TBLB can be a valuable adjunct in the diagnostic process for peripheral lung lesions.

## 5. Limitations

There are some limitations that should be mentioned. First of all, this is a single-center retrospective study, and a limited number of patients were enrolled, limiting the possibility of better statistical analysis such as multivariate regression. However, this is also explained by the novelty of the TB procedure provided by Fujifilm and our protocol, which led to the use of a TB only after a failed CB and not as the first approach technique.

Second, we did not use any kind of additional guidance system as in the other mentioned papers, suggesting that the DY with a TB could further increase with an adjunctive application of more guidance systems, for example, VBN or CBCT.

Third, we used just TBNA needles and biopsy forceps, whereas mini-cryoprobes should be evaluated to compare their DY and to evaluate if DY can further increase by using these novel tools.

However, the use of guidance systems and cryoprobes increases the duration and costs of the procedures. Therefore, this could also be seen as a strength of our study, presenting a novel technique which improves DY without increasing the procedure logistics’ complexity and costs.

Finally, we attributed the non-significant effect of R-EBUS (*p* = 0.68) to biopsy tool stiffness, but operational factors also need to be further investigated; standardized R-EBUS protocols could be a field of interest for future studies.

## 6. Conclusions

The use of a TB can increase DY compared to CB alone, without adding any guidance system or different setting, sedation or biopsy instruments. Its use does not reveal any kind of complications, and the procedure can be easily performed by every well-trained pulmonologist. The diagnostic capability of a TB is particularly increased when the bronchus sign is present, and this could be a starting point for future procedures without referring to advanced bronchoscopic techniques. Therefore, a TB is a useful tool to diagnose PPLs, even when they are potentially resectable. Further studies are needed to evaluate if other related procedures, such as cryobiopsy, can further increase DY with a TB. Furthermore, larger studies are needed to better understand the factors related to better DY in TB use.

## Figures and Tables

**Table 1:** Descriptive statistics of patient and PPL characteristics, numbers of passes and diagnostic yield (DY).

	Overall Population (*n* = 26)	Diagnostic (*n* = 17)	Non-Diagnostic (*n* = 9)	*p*
**Male–*n* (%)**	11 (42)	9 (53)	2 (22)	0.22
**Female–*n* (%)**	15 (58)	8 (47)	7 (78)	
**Age–yr**	76 (63–83)	79 (67–83)	71 (58–82)	0.28
**PPL dimensions (mm)**	29 (17.1–40)	35 (26.6–42)	17.7 (14–28)	0.06
**Number of passes**	3 (3–4.5)	3 (3–5)	3 (3–3)	0.48
**Diagnostic Yield**	17 (65%)			

The table presents number (percentage) of median (quartile 1–3) of the total population who underwent TB for PPLs diagnosis. The population is also presented divided for diagnostic status and the two populations are compared with the Fisher or the Mann–Whitney U-test.

**Table 2:** Peripheral pulmonary nodule (PPL) dimensions, use of R-EBUS, use of TBNA and forceps, and bronchus sign presence divided per group (diagnostic and non-diagnostic specimen).

	Overall Population (*n* = 26)	Diagnostic (*n* = 17)	Non-Diagnostic (*n* = 9)	*p*
**PPL > 20 mm–*n* (%)**	17 (65)	13 (76.5)	4 (44)	0.19
**R-EBUS used–*n* (%)**	14 (54)	10 (59)	4 (44)	0.68
**TBNA needle used–*n* (%)**	3 (11.5)	3 (18)	0	0.53
**Biopsy forceps used–*n* (%)**	24 (92)	15 (88)	9 (100)	0.53
**Bronchus sign present–*n* (%)**	16 (61.5)	13 (76.5)	3 (33)	**0.04**

The table presents the number (percentage) of medians (quartile 1–3) of the total population who underwent a TB procedure for PPL diagnosis. The population is also presented as divided by diagnostic status and the two populations are compared with the Fisher exact test of the Mann–Whitney U-test. Bold number is to highlight the statistically significant difference.

## Data Availability

The data that support the findings of this study are available from the corresponding author, [Lanfranchi F], upon reasonable request.

## Abbreviations

The following abbreviations are used in this manuscript:

PPL	Peripheral pulmonary lesion
NSCLC	Non-small cell lung cancer
TB	Thin bronchoscope
UTB	Ultrathin bronchoscope
CB	Conventional bronchoscopy
CT	Computed tomography
R-EBUS	Radial-probe endobronchial ultrasound
TBLB	Transbronchial lung biopsy
TBNA	Transbronchial needle aspiration
DY	Diagnostic yield
EMN	Electromagnetic navigation
VBN	Virtual bronchoscopic navigation
RB	Robotic bronchoscopy
IHC	Immunohistochemistry
EGFR	Epidermal growth factor receptor
ALK	Anaplastic lymphoma kinase
ROS	Reactive oxygen species
NGS	Next-generation sequencing
IT	Immunotherapy
EFS	Event-free survival
OS	Overall survival
ADK	Adenocarcinoma
SCC	Squamous cell carcinoma
NOS	Carcinoma not otherwise specified
IP	Interventional Pulmonology
GS	Guide sheath
